# Allopurinol and the risk of ventricular arrhythmias in the elderly: a study using US Medicare data

**DOI:** 10.1186/s12916-017-0816-6

**Published:** 2017-03-22

**Authors:** Jasvinder A. Singh, John Cleveland

**Affiliations:** 1 Medicine Service, Birmingham VA Medical Center, Birmingham, AL USA; 2 Department of Medicine at School of Medicine, and Division of Epidemiology at School of Public Health, University of Alabama at Birmingham (UAB), Birmingham, AL USA; 3 Department of Orthopedic Surgery, Mayo Clinic College of Medicine, Rochester, MN USA; 4 University of Alabama at Birmingham, Faculty Office Tower 805B, 510 20th Street S, Birmingham, AL 35294 USA

**Keywords:** Allopurinol, Ventricular arrhythmias, Risk factor, Elderly, Medicare

## Abstract

**Background:**

There are no published human studies investigating whether the use of allopurinol, the most commonly used medication for the treatment of hyperuricemia in gout, the most common type of inflammatory arthritis in adults, has any beneficial effects on ventricular electrophysiology. The objective of our study was to assess whether allopurinol use is associated with a reduction in the risk of ventricular arrhythmias (VA).

**Methods:**

We used the 5% random sample of Medicare beneficiaries from 2006–2012 to examine new allopurinol use and the risk of incident VA. Multivariable Cox regression analyses were adjusted for demographics (age, race, sex), comorbidity, cardiac medications, and conditions associated with VA. We calculated hazard ratios (HR) and 95% confidence intervals (CI).

**Results:**

Of the 28,755 episodes of new allopurinol use, 2538 were associated with incident VA (8.8%). Among patients with incident VA, 54% were male, 78% were White, 75% had gout as the underlying diagnosis, and the mean Charlson–Romano comorbidity score was 4.8. The crude incidence of VA per 1,000,000 person-days declined as the duration of allopurinol use increased: 1–180 days, 151; 181 days to 2 years, 105; and > 2 years, 85. In multivariable-adjusted analyses, compared to non-use, allopurinol use was associated with lower HR of VA of 0.82 (95% CI, 0.76–0.90). Compared to allopurinol non-use, longer allopurinol use durations were significantly associated with lower multivariable-adjusted HR for VA: 1–180 days, 0.96 (95% CI, 0.85–1.08); 181 days to 2 years, 0.76 (95% CI, 0.68–0.85); and > 2 years, 0.72 (95% CI, 0.60–0.87). Multiple sensitivity analyses adjusting for cardiac conditions, anti-arrhythmic drugs and alternate definitions confirmed our findings with minimal/no attenuation of estimates.

**Conclusion:**

Allopurinol use and use duration of more than 6 months were independently associated with a lower risk of VA. Future studies need to assess the pathophysiology of this potential benefit.

**Electronic supplementary material:**

The online version of this article (doi:10.1186/s12916-017-0816-6) contains supplementary material, which is available to authorized users.

## Background

Recent studies have shown that hyperuricemia and gout, a condition with hyperuricemia associated with joint inflammation and/or renal manifestations, are associated with a higher risk of coronary artery disease (CAD), acute cardiovascular events including myocardial infarction (MI) and stroke, and cardiovascular mortality [1–8]. Emerging data suggest that gout and hyperuricemia may also be associated with cardiac arrhythmias such as atrial fibrillation [9–11].

Ventricular arrhythmias (VA) occur  commonly after an acute MI, but are also seen in patients with other cardiac conditions such as valvular or congenital heart disease, cardiomyopathy, hypertension, and other heart diseases [12]. The prevalence of VA (including ventricular tachycardia) in older men and women ranged from 15% to 16% in CAD, 8–9% in hypertension, valvular disease, or cardiomyopathy, and 2–3% in those without cardiac disease [13]; VA is also associated with new coronary events [13]. Therefore, treatment guidelines for VA (and ventricular fibrillation (VF)) emphasize several approaches to reduce associated morbidity and mortality, including immediate treatment using automated external defibrillators in hospitals and the community settings, the use of anti-arrhythmic drugs, and the use of implantable cardioverter-defibrillator, ablation, and revascularization surgery [14, 15].

A recent analysis of the Aspirin Myocardial Infarction Trial that examined mortality rates following daily aspirin administration over 3 years in individuals with documented MI, showed that gout treatments may have had beneficial effects [4]. Compared to MI patients without gout, only MI patients with untreated gout had higher all-cause mortality and CHD mortality, while the risk was not increased in patients with gout treated with gout medications (allopurinol, colchicine, or probenecid) [4]. An observational study reported that allopurinol use was associated with a reduction of the risk of incident atrial fibrillation in the elderly [16]. Together, these data raise an important question: Can allopurinol use reduce the risk of VA?

Animal study data suggest that allopurinol may prevent VA. In both rat and guinea pig models of ischemia-reperfusion injury, allopurinol treatment reduced the incidence of VA, VF, and possibly mortality [17–19]. This efficacy correlated with significant attenuation of reperfusion-induced transmural conduction delay [18]. Proposed mechanisms for allopurinol’s anti-arrhythmic action include (1) an anti-oxidant action [20], via endothelial nitric oxide synthase reduction [21] that likely underlies the noted improvement of endothelial function [22–25]; (2) an anti-ischemic action [26] and associated reduction in blood pressure [27, 28]; and (3) the reduction of left ventricular mass [29, 30] and pressure overload [31]. Given this basic science discovery, our recent finding of a beneficial effect of allopurinol use on the risk of atrial fibrillation [16], and findings of mortality reduction with gout treatment after MI in the Aspirin Myocardial Infarction Trial [4], we hypothesized that allopurinol use will be associated with a reduction in the risk of VA.

To our knowledge, there are no published human studies addressing this question. Therefore, we aimed to assess, whether (1) allopurinol use was associated with a lower risk/hazard of VA; and (2) allopurinol use duration was associated with a lower risk/hazard of VA. In an exploratory analysis, we assessed whether the risk reduction in VA with allopurinol use varied by previous MI, and other cardiac conditions that are risk factors for VA, and explored the association of allopurinol with the risk of VF.

## Methods

### Study cohort and the population of interest

We conducted a retrospective cohort study using a 5% random sample of persons who were Medicare beneficiaries at any point in 2006–2012, using the same dataset and a similar protocol to a study previously published [16]. These data were obtained from the Centers for Medicare and Medicaid Services Chronic Condition Data Warehouse. The Medicare 5% random sample file contains all insurance claims for each beneficiary and has been widely used for epidemiological research [32, 33]. We abstracted the following data from each of the respective files: (1) a beneficiary summary file which has demographic information, birthdate, death date, sex, race, and monthly entitlement indicators (A/B/C/D); (2) a part D file, which has information on prescription claims, dose, supply, drug name; and (3) inpatient and outpatient claim files, which contain the diagnosis codes for each claim and claim dates. In order to be eligible, beneficiaries had to reside in the US from 2006–2012, be enrolled in Medicare fee-for-service with pharmacy coverage (Parts A, B, and D) and not enrolled in a Medicare Advantage Plan, and receive a new treatment with allopurinol (see following section for definition). The Institutional Review Board at the University of Alabama at Birmingham approved the study; informed consent was waived as this was a database analysis.

### Allopurinol treatment definition

A beneficiary began an allopurinol treatment episode by filling an allopurinol prescription, provided they had not filled an allopurinol prescription in the previous 365 days. Days of exposure were calculated based on the days supply variable provided in the Medicare Part D file and included a 30 day residual period. For example, if a patient received a 90-day supply, then we considered them exposed for 120 days; 90 days of supply plus 30 days of residual biological effect. The purpose was two-fold, first to capture inconsistent medication adherence, and second to account for any residual protective biologic effects of the medication itself. If the person filled another prescription prior to the end of the 30-day residual period, then we considered this as one continuous treatment episode and a new 30-day residual period would begin when the supply for the second prescription ran out. If the person did not fill another prescription, then the first allopurinol treatment episode would end and a second episode would not commence until the next filled prescription. We defined allopurinol treatment duration as “none”, “1–180 days”, “181 days to 2 years”, and “>2 years”, as determined a priori to reflect short, intermediate and long-term use, similar to a previous study [16]. Subjects contributed to the “none” category during periods where they were not in an allopurinol treatment episode.

### Study covariates and potential confounders

Study covariates and potential confounders included age, sex, race, common cardiac medications (statins, diuretics, ACE inhibitors, beta-blockers), aspirin and specific anti-arrhythmic drugs (digoxin, calcium channel blockers, amiodarone, flecainide, and ranolazine), common conditions associated with ventricular arrhythmias (CAD, cardiomyopathy-dilated or hypertrophic, congestive heart failure, congenital heart disease, valvular heart disease, renal failure, dialysis, sarcoidosis, hyperkalemia), and Charlson–Romano comorbidity index score, a valid measure of medical comorbidity [34].

### Study outcome

The outcome of interest was the occurrence of incident VA. Beneficiaries were required to have no diagnosis of VA in a 365-day baseline period before the initiation of allopurinol use. Incident VA after the initiation of a new allopurinol prescription was identified based on the occurrence of the International Classification of Diseases, ninth revision, common modification (ICD-9-CM) codes (427.1, 427.2, 427.4x, 427.5, 427.60, or 427.69) in the Medicare claims, a modified list of codes based on a validated approach with a positive predictive value of 92% to 100% [35, 36].

Eligible beneficiaries were followed beginning on the earliest allopurinol treatment date in the study period and ending on first occurrence of losing full Medicare coverage, VA diagnosis, death, or end of study period (December 31, 2012). If a beneficiary lost and regained Medicare coverage during the study period, then they were eligible to reenter and contribute more treatment episodes. Patients could contribute multiple allopurinol treatment episodes during different time periods. Summary statistics were assessed for patients with and without incident VA. We calculated crude incident rates of VA by allopurinol use (yes vs. no) and the duration of allopurinol use.

### Statistical analyses

The main analysis assessed the association of allopurinol use and incident VA, and the duration of allopurinol use and incident VA, analyzed using separate Cox proportional hazards regression models. We performed univariate and multivariable-adjusted analyses, accounting for important covariates and confounders listed in the section above (demographics, comorbidity, common cardiac medications, and conditions). To account for the correlation due to patients contributing multiple treatment episodes, we used the Huber–White “Sandwich” variance estimator to calculate robust standard errors for the parameter estimates [37]. We calculated hazard ratios (HR) and 95% confidence intervals (CI).

Sensitivity analyses were conducted for allopurinol use and the duration of allopurinol use by (1) replacing the Charlson–Romano score with specific risk factors for VA (conditions associated with VA; sensitivity analysis 1); (2) replacing the Charlson–Romano score with specific risk factors for VA (as in previous sensitivity analyses), and adjusting for aspirin and anti-arrhythmic drugs (digoxin, calcium channel blockers, amiodarone, flecainide, and ranolazine; sensitivity analysis 2); (3) using a different set of ICD-9 codes (427.1, 427.2, 427.4, 427.41, 427.42, 427.5, 798, 798.1, and 798.2) based on the study by Hennessey et al. [35] (sensitivity analysis 3); and (4) adjusting additionally for anti-arrhythmic medications (mexilitine, propafenone and dofetilide; sensitivity analysis 4). We considered other anti- arrhythmic drugs, including quinidine and procainamide, but a low frequency of use precluded their inclusion in the model.

Subgroup analyses were performed for each of the specific disease risk factors for VA, and by the history of previous MI to better understand whether the risk reduction varies by the underlying etiology for VA or by prior MI. We analyzed MI separately, since MI is a discrete event that has a well-established association with VA, which is associated with significant morbidity and mortality. We anticipated much fewer events for VF (ICD-9 code, 427.4, 427.41 and 427.42), and anticipating lower power, we planned an exploratory analysis for this outcome. We also performed subgroup analyses by the underlying diagnosis (gout vs. non-gout) to assess differential benefit by diagnosis and for patients who were not receiving anti-arrhythmic or cardio-protective drugs, to assess the protective effect associated with xanthine oxidase inhibition with allopurinol.

## Results

### Clinical and demographic characteristics

**Fig. 1:**
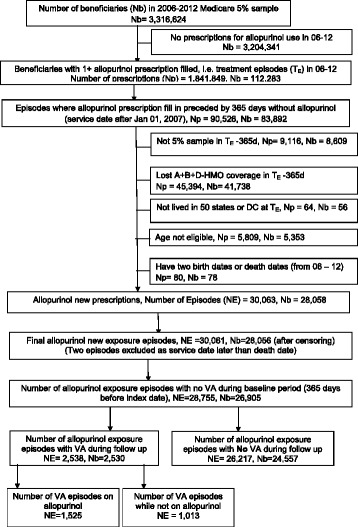
Patient selection flow chart. The flow chart shows the selection of new allopurinol exposure episodes after applying all the eligibility criteria, including an absence of VA and the absence of any allopurinol filled prescription in the baseline period of 365 days (new user design). We found 28,755 new allopurinol exposure episodes in 26,905 patients. Of these, 2538 ended in incident VA and 26,217 ended without incident VA. * We followed each eligible patient with a new filled allopurinol prescription until the patient lost full Medicare coverage, had VA (the outcome of interest), died or reached the each of the study period on December 31, 2012, whichever came first. For some of these patients, the VA occurred on days covered by allopurinol exposure (n = 1525), yet other patients had periods of no allopurinol exposure after an initial qualifying allopurinol exposure during which VA occurred (n = 1013). *Nb* Number of beneficiaries, *T*_*E*_ treatment episodes, *Np* Number of allopurinol prescriptions, *NE* Number of qualified episodes of new allopurinol prescriptions, *VA* Ventricular arrhythmia

The study flow chart is shown in Fig. 1. Of the 28,755 episodes of new allopurinol use, 2538 were associated with incident VA during the follow-up (8.8%). Allopurinol daily dose was < 200 mg/day in 46%, 200–299 mg/day in 18%, and ≥ 300 mg/day in 36%. The underlying diagnosis was gout in 74%, asymptomatic hyperuricemia in 5%, renal calculi in 2%, and other diagnoses in 19%. Of these, 1525 VA episodes occurred during days of allopurinol exposure and 1013 occurred during periods of no allopurinol exposure. Among patients with incident VA, 54% were male, 78% were White, 40% were in Southern USA, and the mean Charlson–Romano comorbidity score was 4.8 (Table 1). We noted a significant difference in age, sex, race, Charlson–Romano scores, and region between VA and non-VA groups (Table 1). The crude incidence of ventricular arrhythmias/1,000,000 person-days declined as the duration of allopurinol use increased: 1–180 days, 151; 181 days to 2 years, 105; and > 2 years, 85 (Additional file 1: Appendix 1).

**Table 1:** Demographic and clinical characteristics of episodes of new allopurinol users (baseline with no ventricular arrhythmias; baseline was 365 days)

	All new allopurinol use episodes	Allopurinol new use episodes^a^ associated with versus without ventricular arrhythmias (VA) during the follow-up		*P* value
		VA	No VA	
**Total, N (episodes)**	**28,755**	**2538**	**26,217**	
Age, mean (SD)	76.6 (7.4)	77.2 (7.3)	76.5 (7.5)	**< 0.0001**
Sex, N (%)				**< 0.0001**
Male	14,074 (48.9%)	1369 (53.9%)	12,705 (48.5%)	
Female	14,681 (51.1%)	1169 (46.1%)	13,512 (51.5%)	
Race/Ethnicity, N (%)				**< 0.0001**
White	22,693 (78.9%)	1976 (77.9%)	20,717 (79.0%)	
Black	3510 (12.2%)	382 (15.1%)	3128 (11.9%)	
Hispanic	606 (2.1%)	49 (1.9%)	557 (2.1%)	
Asian	1268 (4.4%)	89 (3.5%)	1179 (4.5%)	
Native American	97 (0.3%)	7 (0.3%)	90 (0.3%)	
Other/unknown	581 (2.0%)	35 (1.4%)	546 (2.1%)	
Region, N (%)				**0.03**
Northeast	4607 (16.0%)	460 (18.1%)	4147 (15.8%)	
Midwest	7315 (25.4%)	626 (24.7%)	6689 (25.5%)	
South	11,563 (40.2%)	1003 (39.5%)	10,560 (40.3%)	
West	5270 (18.3%)	449 (8.5%)	4821 (18.4%)	
Charlson–Romano comorbidity score	3.65 (3.25)	4.79 (3.40)	3.54 (3.21)	**< 0.0001**

SD, standard deviation

^a^All data are at episode level

Significant *P* values are in bold

### Association of allopurinol use with ventricular arrhythmias

In multivariable-adjusted analyses, compared to allopurinol non-use, allopurinol use was associated with significantly lower HR of 0.82 of VA (95% CI, 0.76–0.90), as were allopurinol use durations of > 6 months: 1–180 days, 0.96 (95% CI, 0.85–1.08); 181 days to 2 years, 0.76 (95% CI, 0.68–0.85); and > 2 years, 0.72 (95% CI, 0.60–0.87) (Model 1; Table 2). Other factors associated with significantly higher hazard ratios of VA are shown in Table 2.

**Table 2:** Association of risk factors with hazard of ventricular arrhythmias in patients who received allopurinol with no baseline ventricular arrhythmias before the index date of allopurinol episode

	Univariate		Multivariable adjusted (Model 1)		Multivariable adjusted (Model 2)	
	HR (95% CI)	*P* value	HR (95% CI)	*P* value	HR (95% CI)	*P* value
Age (in years)						
65 to < 75	Ref		Ref		Ref	
75 to < 85	**1.25 (1.15–1.36)**	**< 0.0001**	**1.19 (1.09–1.30)**	**< 0.0001**	**1.19 (1.09–1.30)**	**< 0.0001**
≥ 85	**1.43 (1.28–1.60)**	**< 0.0001**	**1.37 (1.22–1.53)**	**< 0.0001**	**1.37 (1.22–1.53)**	**< 0.0001**
Sex						
Male	Ref		Ref		Ref	
Female	0.81 (0.75–0.87)	0.50	**0.74 (0.68–0.80)**	**< 0.0001**	**0.73 (0.68–0.80)**	**< 0.0001**
Race						
White	Ref		Ref		Ref	
Black	**1.32 (1.19–1.48)**	**< 0.0001**	**1.30 (1.16–1.45)**	**< 0.0001**	**1.29 (1.15–1.44)**	**< 0.0001**
Other	**0.80 (0.69–0.94)**	**0.005**	**0.78 (0.67–0.91)**	**0.002**	**0.78 (0.67–0.90)**	**0.001**
Charlson–Romano score, per unit change	**1.15 (1.14–1.16)**	**< 0.0001**	**1.14 (1.13–1.16)**	**< 0.0001**	**1.14 (1.13–1.16)**	**< 0.0001**
Diuretics	1.12 (0.94–1.33)	0.21	1.03 (0.86–1.24)	0.72	1.03 (0.86–1.24)	0.74
Statins	0.86 (0.70–1.05)	0.13	**0.79 (0.64–0.97)**	**0.02**	**0.79 (0.64–0.97)**	**0.02**
ACE inhibitor	1.09 (0.88–1.34)	0.43	1.11 (0.90–1.38)	0.32	1.11 (0.90–1.38)	0.33
Beta blockers	**1.42 (1.20–1.68)**	**< 0.0001**	**1.41 (1.18–1.68)**	**0.0001**	**1.40 (1.18–1.68)**	**0.0001**
Allopurinol use	**0.86 (0.79–0.94)**	**0.001**	**0.82 (0.76–0.90)**	**< 0.0001**	–	–
Allopurinol use duration^a^						
0 days	Ref		–	–	Ref	
1 to 180 days	0.99 (0.88–1.12)	0.88	–	–	0.96 (0.85–1.08)	0.49
181 days to 2 years	**0.81 (0.72–0.90)**	**0.0002**	–	–	**0.76 (0.68–0.85)**	**< 0.0001**
> 2 years	**0.74 (0.62–0.90)**	**0.002**	–	–	**0.72 (0.60–0.87)**	**0.001**

Significant hazards ratios and *P* values are in bold

^a^Based on person day count

Model 1 = Allopurinol use + age + race + sex + Charlson–Romano score + beta blockers + diuretics + ACE inhibitors + statins

Model 2 = Allopurinol duration + age + race + sex + Charlson–Romano score + beta blockers + diuretics + ACE inhibitors + statins

*HR* hazard ratio, *CI* confidence interval, *Ref* referent category

Several multivariable-adjusted hierarchical sensitivity analyses confirmed the main study findings with no/minimal change in estimates or the level of significance, including adjustment for diseases that are known risk factors for VA instead of Charlson index score (sensitivity analysis 1; Table 3); additional adjustment for aspirin, digoxin, calcium channel blockers, amiodarone, flecainide, and ranolazine revealed the same hazard ratio as in previous analysis (sensitivity analysis 2; data not shown). Rerunning these models with a different set of ICD-9 codes for VA based on the study by Hennessey et al. [35] (sensitivity analysis 3; Additional file 1: Appendix 2) or additional adjustment for anti-arrhythmic medications, mexilitine, propafenone, and dofetilide (sensitivity analysis 4; Additional file 1: Appendix 3), confirmed the main results.

**Table 3:** Sensitivity Analysis 1: Association of risk factors with hazard of ventricular arrhythmias adjusted for specific disease risk factors for ventricular arrhythmias instead of Charlson index

	Multivariable adjusted (Model 3)		Multivariable adjusted (Model 4)	
	HR (95% CI)	*P* value	HR (95% CI)	*P* value
Allopurinol use	**0.82 (0.75–0.89)**	**< 0.0001**	–	–
Allopurinol use duration^a^				
0 days (non-use)	–	–	Ref	
1–180 days	–	–	0.92 (0.82–1.04)	0.18
181 days to 2 years	–	–	**0.76 (0.68–0.84)**	**< 0.0001**
> 2 years	–	–	**0.78 (0.65–0.94)**	**0.009**

^a^Based on person day count

Model 3 = Allopurinol use + age + race + sex + beta blockers + diuretics + ACE inhibitors + statins + CAD + cardiomyopathy + heart failure + congenital heart disease + valvular heart disease + renal failure + dialysis + sarcoidosis + hyperkalemia

Model 4 = Allopurinol duration + age + race + sex + beta blockers + diuretics + ACE inhibitors + statins + CAD + cardiomyopathy + heart failure + congenital heart disease + valvular heart disease + renal failure + dialysis + sarcoidosis + hyperkalemia

Significant hazards ratios and *P* values are in bold

*HR* hazard ratio, *CI* confidence interval, *Ref* referent category

### Exploratory subgroup analyses by VA risk factors, previous MI, race/sex, and for VF

We found that allopurinol-VA associations were similar in cohorts with and without each VA risk factor with three exceptions, i.e., hazard-reduction was slightly more in patients without CAD, heart failure, or dialysis compared to patients with each respective condition (Table 4); differences in statistical significance was likely due to sample size differences between groups with versus without each condition.

**Table 4:** Exploratory subgroup analysis of the main analyses: multivariable-adjusted hazard ratio of ventricular arrhythmias (VA) for allopurinol use and for the duration of allopurinol use by each VA risk factor

	Allopurinol use (using model 5) HR (95% CI) [*P* value]		Allopurinol use duration (using model 6) HR (95% CI) [*P* value]			
	No	Yes	0 day	1 to 180 days	181 days to 2 years	> 2 years
Coronary artery disease						
No	1	**0.60 (0.47–0.78) [0.0001]**	Ref	0.71 (0.47–1.06) [0.09]	**0.57 (0.42–0.83) [0.002]**	**0.45 (0.25–0.81) [0.007]**
Yes	Ref	**0.85 (0.77–0.95) [0.003]**	Ref	0.97 (0.84–1.12) [0.64]	**0.79 (0.69–0.90) [0.001]**	**0.79 (0.62–0.99) [0.04]**
Cardiomyopathy-dilated or hypertrophic						
No	Ref	**0.80 (0.71–0.90) [0.0002]**	Ref	0.91 (0.77–1.07) [0.26]	**0.75 (0.65–0.88) [0.0002]**	**0.71 (0.55–0.91) [0.007]**
Yes	Ref	**0.84 (0.71–0.99) [0.04]**	Ref	0.95 (0.75–1.21) [0.68]	**0.77 (0.62–0.96) [0.02]**	0.73 (0.46–1.15) [0.17]
Heart failure						
No	Ref	**0.74 (0.65–0.85) [< 0.0001]**	Ref	0.88 (0.73–1.07) [0.20]	**0.67 (0.55–0.81) [< 0.0001]**	**0.66 (0.50–0.89) [0.006]**
Yes	Ref	0.89 (0.78–1.01) [0.08]	Ref	0.98 (0.81–1.18) [0.82]	**0.84 (0.74–0.99) [0.04]**	0.79 (0.57–1.10) [0.16]
Congenital heart disease						
No	Ref	**0.82 (0.74–0.90) [< 0.0001]**	Ref	0.93 (0.81–1.06) [0.26]	**0.76 (0.68–0.87) [< 0.0001]**	**0.73 (0.59–0.91) [0.005]**
Yes	Ref	0.80 (0.41–1.57) [0.52]	Ref	1.36 (0.60–3.11) [0.47]	0.52 (0.18–1.44) [0.21]	**0.00 (0.0–0.0) [< 0.0001]**
Valvular heart disease						
No	Ref	**0.82 (0.74–0.92) [0.0004]**	Ref	0.94 (0.81–1.09) [0.39]	**0.77 (0.67–0.88) [0.0002]**	**0.74 (0.58–0.94) [0.01]**
Yes	Ref	**0.78 (0.63–0.97) [0.025]**	Ref	0.92 (0.68–1.24) [0.56]	**0.72 (0.58–0.95) [0.018]**	0.66 (0.38–1.13) [0.13]
Renal failure						
No	Ref	**0.81 (0.67–0.98) [0.03]**	Ref	0.93 (0.70–1.24) [0.62]	0.79 (0.62–1.01) [0.06]	0.63 (0.42–0.96) [0.03]
Yes	Ref	**0.81 (0.73–0.91) [0.0003]**	Ref	0.93 (0.80–1.09) [0.37]	**0.75 (0.65–0.86) [< 0.0001]**	**0.75 (0.58–0.97) [0.03]**
Dialysis						
No	Ref	**0.81 (0.73–0.89) [< 0.0001]**	Ref	0.90 (0.79–1.03) [0.14]	**0.76 (0.67–0.87) [< 0.0001]**	**0.73 (0.53–0.90) [0.004]**
Yes	Ref	1.36 (0.54–3.43) [0.52]	Ref	**2.43 (1.03–5.69) [0.04]**	0.73 (0.19–2.77) [0.64]	**0.00 (0.0–0.0) [< 0.0001]**
Sarcoidosis						
No	Ref	**0.81 (0.74–0.89) [< 0.0001]**	Ref	0.93 (0.81–1.06) [0.29]	**0.76 (0.67–0.85) [< 0.0001]**	**0.72 (0.58–0.90) [0.003]**
Yes	Ref	0.86 (0.13–5.58) [0.88]	Ref	0.73 (0.05–11.2) [0.82]	1.11 (0.11–11.41) [0.93]	**0.00 (0.0–0.0) [< 0.0001]**
Hyperkalemia						
No	Ref	**0.81 (0.73–0.91) [0.0002]**	Ref	0.92 (0.78–1.08) [0.31]	**0.76 (0.66–0.88) [0.0001]**	**0.73 (0.57–0.93) [0.01]**
Yes	Ref	**0.82 (0.68–0.99) [0.04]**	Ref	0.95 (0.73–1.22) [0.68]	**0.76 (0.59–0.97) [0.03]**	0.67 (0.42–1.08) [0.10]

Model 5 = Allopurinol use + age + race + sex + beta blockers + diuretics + ACE inhibitors + statins + CAD + cardiomyopathy + heart failure + congenital heart disease + valvular heart disease + renal failure + dialysis + sarcoidosis + hyperkalemia + aspirin + digoxin + calcium channel blockers + amiodarone + flecainide + ranolazine

Model 6 = Allopurinol duration + age + race + sex + beta blockers + diuretics + ACE inhibitors + Statins + CAD + cardiomyopathy + heart failure + congenital heart disease + Valvular heart disease + renal failure + dialysis + sarcoidosis + hyperkalemia + aspirin + digoxin + calcium channel blockers + amiodarone + flecainide + ranolazine

Significant hazards ratios and *P* values are in bold

**Fig. 2:**
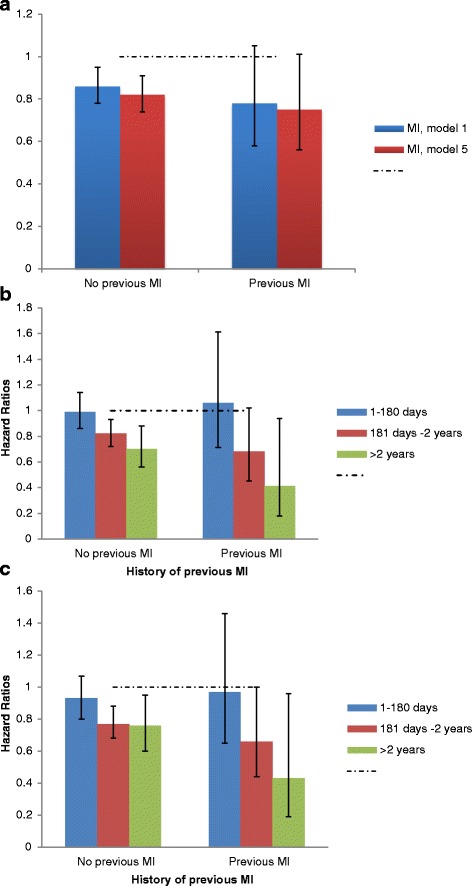
Examining the effect of previous myocardial infarction (MI) on the associations of allopurinol use (2a) and allopurinol use duration (2b, 2c) with incident ventricular arrhythmias (VA). **a** Association of allopurinol use with VA by previous MI: Models 1 and 5. **b** Association of allopurinol use duration with VA by previous MI: Model 2. **c** Association of allopurinol use duration with VA by previous MI: Model 6. Each solid bar represents the hazard ratio estimate for allopurinol use (vs. non-use) for both Models 1 and 5 (panel **a**) or allopurinol use duration for Model 2 (panel **b**; multivariable model adjusted for demographics, Charlson–Romano score, beta blockers, diuretics, ACE inhibitors and statins) and model 6 (panel **c**; multivariable model adjusted for demographics, beta blockers, diuretics, ACE inhibitors, statins, VA risk factor conditions, aspirin, digoxin, calcium channel blockers, amiodarone, flecainide, and ranolazine), each panel given the presence or absence of previous MI. A hazard ratio of 1.0 represents the reference hazard with no exposure to allopurinol. Error bars represent the 95% confidence interval for each hazard ratio and inclusion of 1.0 in this range indicates that the hazard ratio is not significant

The hazard ratios for allopurinol use for VA were similar in patients with versus without previous MI, but the results for allopurinol use were not significant in those with previous MI (Additional file 1: Appendix 4; Fig. 2). We found that longer allopurinol use durations, especially allopurinol use for over 2 years, were significantly associated with lower HR for VA in both patients with or without previous MI, and hazard reduction was more impressive in patients with previous MI (Additional file 1: Appendix 4; Fig. 2). Black race and male sex were associated with higher hazard of VA versus counterparts (Additional file 1: Appendix 5).

We observed 245 episodes of VF during follow up, 96 were during allopurinol exposed days and 149 were not; we noted 29,730 allopurinol episodes without VF. In an exploratory multivariable-adjusted analysis of VF we found that Charlson–Romano score was the only covariate significantly associated with an increased hazard of VA, while allopurinol use or use duration were not significantly associated (Additional file 1: Appendix 6).

### Exploratory subgroup analyses by diagnosis and in patients not receiving anti-arrhythmic or cardio-protective drugs

Allopurinol use reduced the hazard of VA in patients with gout, HR was 0.81 (95% CI, 0.73–0.90); hazard reduction in those without gout (a much smaller sample) was not significant, 0.89 (95% CI, 0.71–1.10) (Additional file 1: Appendix 7). Allopurinol use duration was significantly associated with reduction of hazard of VA in patients with gout, similar to the main analysis (Additional file 1: Appendix 7). In analyses limited to patients not receiving anti-arrhythmic or cardio-protective drugs, allopurinol use and use duration were associated significantly with the hazard of VA, as in the main analysis (Additional file 1: Appendix 8).

## Discussion

In this study of a nationally representative sample of older Americans, we made several novel observations. We found that allopurinol use was independently associated with a lower hazard of incident VA. We also found that, compared to allopurinol non-use, allopurinol use durations greater than 6 months were associated with significant reduction in the hazard of incident VA. Associations of allopurinol use and use duration with hazard reduction of VA were confirmed in patients with gout, and for patient subgroups who were  not on anti-arrhythmic or cardio-protective drugs. The hazard reduction of VA with allopurinol differed by the history of previous MI and the presence of other diseases, which are known risk factors for VA. These novel study findings deserve further elaboration and discussion.

Recent experimental evidence using animal models of ischemia-reperfusion injury showed that treatment with allopurinol reduced the incidence of VA and possibly mortality [17–19]. To our knowledge, this is the first study in humans to show that, compared to non-use, allopurinol use was associated with a 18% reduction (clinically relevant) in the hazard of incident VA and longer durations of allopurinol use of 181 days to 2 years and more than 2 years were associated with hazard reductions of 24% and 28%, respectively (Table 3). In the absence of prior studies in humans, no comparisons could be done. Animal studies support allopurinol’s anti-arrhythmic action in ischemia-reperfusion injury models [17–19], including a placebo-controlled study of allopurinol [17]. The mechanism of hazard reduction of VA with allopurinol is unknown. We speculate that it may be related to the significant attenuation of reperfusion-induced transmural conduction delay, as noted with allopurinol use in a guinea pig ischemia-reperfusion injury model [18]. Other proposed mechanisms may be related to other noted beneficial effects of allopurinol, including associated anti-oxidant action [20, 21], an anti-ischemic action [26], blood pressure reduction [27, 28], improvement of endothelial function [22–25], and reduction of left ventricular mass [29, 30]. Some of these processes may be sequential and many are likely on the causative pathway of VA.

VA often occur in patients with CAD and cardiac dysfunction, including heart failure [13]. The generation of reactive oxygen species in these disorders can contribute to induction of arrhythmias, via multiple mechanisms, including the alteration of cardiac ionic channels [38] and cardiac cell death associated ventricular dysfunction [39]. Oxidative-stress mediated tissue injury during ischemia and reperfusion may be associated with both ischemia and reperfusion-induced arrhythmias [17]. Xanthine oxidase has been implicated in cardiovascular disease and xanthine oxidase inhibition for its treatment [24, 40–42].

Allopurinol inhibits xanthine oxidase activity, which in turn inhibits superoxide radical production and reduces oxidative stress [43]. Allopurinol’s anti-oxidant effect likely leads to an improvement in endothelial function and possibly has an anti-ischemic effect, which might prevent left ventricular hypertrophy. In particular, both the endothelial function improvement [22–25], and the anti-ischemic effects [26] associated with the use of allopurinol may be the key mechanisms related to this anti-arrhythmic effect.

Our observation may have practical implications, although they need further confirmation before a widespread implementation. Allopurinol is a well-known effective, affordable treatment for gout, the most common inflammatory arthritis in adults, affecting 5% of adult Americans [44]. The emerging evidence of cardioprotective action of allopurinol provides a greater urgency for optimal use of allopurinol in all patients with gout (except rare instance). This observation of anti-arrhythmic action may lead to a preference of allopurinol over other urate-lowering agents in gout and related conditions. It is possible that there is an even greater advantage of allopurinol use in patients with gout and a concomitant well-known pro-arrhythmic condition. Our findings indicate that there may be a potential role for allopurinol as a xanthine-oxidase inhibitor beyond the joint, including a protective role in preventing VAs in patients with myocardial ischemia and damage.

The estimates for allopurinol use and use duration with VA were robust and were unaltered in multiple sensitivity analyses. This observation of an anti-arrhythmic effect is similar to the recent observations of potentially cardioprotective effects of allopurinol in the elderly [16, 45, 46]. Longer duration of allopurinol use was associated with more VA hazard reduction, representing a dose effect with a magnitude similar to that noted with the associated reduction of myocardial infarction, stroke, or atrial fibrillation [16, 45, 46].

An interesting observation was that the beneficial effect of allopurinol use was similar for subgroup analyses for most diseases that are risk factors for VA, including valvular heart disease, congenital heart disease, heart failure, renal failure, dialysis, cardiopmyopathy, and hyperkalemia. Minor differences in statistical significance seemed related to a smaller sample size for patients with each VA risk factor. One interesting observation was that, although beneficial for both, allopurinol’s beneficial effect was greater in magnitude for patients without CAD versus with CAD, 38% versus 13% hazard reduction (model 1). Allopurinol use was associated with statistically significant hazard reduction for VA (18%) in patients without previous MI, but reduction was not statistically significant in those with previous MI (Model 5). This is not surprising since ischemia-reperfusion injury is one of the best-described pathophysiologic associations between MI and VA [17–19, 47–50]. Irreversible structural damage may already have occurred in some patients with CAD/MI, making allopurinol not as effective in patients with CAD/MI. This finding may have important implications, if confirmed in other studies. Additionally, a smaller sample size for patients with MI may have made this analysis underpowered.

We noted an association of older age and Black race with a higher hazard of VA, confirming a similar previous finding [51–53]. We also noted that older age was no longer significantly associated with the risk of VA once the model was adjusted for VA risk factors, such as CAD, congenital heart disease, renal failure, and others. This indicated that age-VA associations were not due to chronological age but rather reflected the higher risk imparted due to specific diseases that are VA risk factors, more common in the elderly.

Study findings must be interpreted considering their strengths and limitations. An observational study design makes our findings susceptible to confounding bias. We tried to reduce confounding bias by including several important patient characteristics, common cardiac medications and adjusting for risk factor conditions for VA. We used ICD-9 diagnostic codes from Medicare claims for the assessment of VA, which makes results liable to misclassification bias; this likely biased our results towards null. However, similar approaches have been shown to have high positive predictive values in a systematic review of claims-based definitions of ventricular arrhythmias [36]. Sensitivity analysis using the VA code algorithm with the highest positive predictive value (92–100%) reproduced the same result as our main analysis [35]. We realized a priori that there would be insufficient number of VF events, making this analysis underpowered and therefore exploratory in nature, as shown. Limited sources and expected few data for febuxostat (regulatory approval in 2009; another xanthine oxidase inhibitor) prevented us from performing additional comparative effectiveness studies comparing febuxostat to allopurinol.

The main strength of this study lies in the potential for generalization to the elderly US population and to all allopurinol new-users, regardless of the underlying diagnosis. Our study had a large sample size and adequate number of outcome events. Another strength is the incident (or new) user design, which reduces bias by avoiding adjustment for characteristics that may be in the causal pathway and allows capture of both early and late events [54], which is important given the study objectives. We controlled for several potential confounders (risk factors for VA; cardiac medications) to reduce bias and conducted multiple sensitivity analyses, which confirmed the robustness of our findings.

## Conclusions

In conclusion, we found a significant association between incident allopurinol use and a lower hazard of VA in the elderly. This result was more pronounced for longer allopurinol use durations. Results for patients with an underlying diagnosis of gout were similar to the entire sample of allopurinol users. We also found some differences in VA risk reduction with allopurinol in patients with and without CAD and previous MI. Mechanisms of reduction of ventricular arrhythmias with allopurinol need to be examined in future studies. Future studies should also examine the underlying mechanisms for why the VA hazard reduction with allopurinol varies by CAD and previous MI.

## Additional file

Additional file 1: Appendix 1. Crude incidence rate of ventricular arrhythmias with allopurinol exposure. Appendix 2. Sensitivity analysis 3: Main models run using a different set of ICD-9 codes for ventricular arrhythmias* based on the study by Hennessey et al. [38]. Appendix 3. Sensitivity analysis 4: Association of risk factors with hazard of ventricular arrhythmias in patients who received allopurinol including specific disease risk factors and three additional anti-arrhythmic medications* (mexilitine, propafenone and dofetilide). Appendix 4. Subgroup analysis by prior myocardial infarction (MI): Allopurinol use and duration of allopurinol use models by prior MI diagnosis. Appendix 5. Hazard ratios of ventricular arrhythmias by race and sex, adjusted for other factors. Appendix 6. Association of risk factors with hazard of ventricular fibrillation in patients who received allopurinol with no baseline ventricular fibrillation before the index date of allopurinol episode. Appendix 7. Sensitivity analysis by underlying diagnosis of gout versus non-gout: association of allopurinol with hazard of ventricular arrhythmias adjusted for specific disease risk factors for ventricular arrhythmias. Appendix 8. Sensitivity analysis limiting the cohort to patients not receiving any anti-arrhythmic or cardio-protective drugs: Association of allopurinol with hazard of ventricular arrhythmias adjusted for specific disease risk factors for ventricular arrhythmias. (DOCX 58 kb)

## Abbreviations

CAD: coronary artery disease
CI: confidence interval
HR: hazard ratio
ICD-9-CM: international classification of diseases, ninth revision, common modification
MI: myocardial infarction
VA: ventricular arrhythmia
VF: ventricular fibrillation
