# Supplementary material for: Allopurinol and the risk of ventricular arrhythmias in the elderly: a study using US Medicare data
Source: BMC Med. 2017 Mar 22;15:59. doi: 10.1186/s12916-017-0816-6 (PMC5361697; doi:10.1186/s12916-017-0816-6)
Supplement: Additional file 1: — Appendix 1. Crude incidence rate of ventricular arrhythmias with allopurinol exposure. Appendix 2. Sensitivity analysis 3: Main models run using a different set of ICD-9 codes for ventricular arrhythmias* based on the study by Hennessey et al. [38]. Appendix 3. Sensitivity analysis 4: Association of risk factors with hazard of ventricular arrhythmias in patients who received allopurinol including specific disease risk factors and three additional anti-arrhythmic medications* (mexilitine, propafenone and dofetilide). Appendix 4. Subgroup analysis by prior myocardial infarction (MI): Allopurinol use and duration of allopurinol use models by prior MI diagnosis. Appendix 5. Hazard ratios of ventricular arrhythmias by race and sex, adjusted for other factors. Appendix 6. Association of risk factors with hazard of ventricular fibrillation in patients who received allopurinol with no baseline ventricular fibrillation before the index date of allopurinol episode. Appendix 7. Sensitivity analysis by underlying diagnosis of gout versus non-gout: association of allopurinol with hazard of ventricular arrhythmias adjusted for specific disease risk factors for ventricular arrhythmias. Appendix 8. Sensitivity analysis limiting the cohort to patients not receiving any anti-arrhythmic or cardio-protective drugs: Association of allopurinol with hazard of ventricular arrhythmias adjusted for specific disease risk factors for ventricular arrhythmias. (DOCX 58 kb) [file 12916_2017_816_MOESM1_ESM.docx]

**Additional file 1**

**Appendix 1.** Crude Incidence rate of Ventricular arrhythmias with allopurinol exposure

|  | Person-days of follow up | #Cases of Ventricular arrhythmias | Ventricular arrhythmias Incidence Rate  per 1,000,000 person-days |
| --- | --- | --- | --- |
| Allopurinol exposure* |  |  |  |
| Yes | 12,413,938 | 1,525 | 123 |
| No | 7,999,359 | 1,013 | 127 |
|  |  |  |  |
| Allopurinol use duration |  |  |  |
| 0 days | 7,999,359 | 1,013 | 127 |
| 1 to 180 days | 5,726,945 | 860 | 151 |
| 181 days to 2 years | 4,932,388 | 517 | 105 |
| >2 years | 1,754,605 | 148 | 85 |

*Allopurinol exposure up to 30 days after last day of medication fill/refill; baseline period for allopurinol was 365 days, i.e., each new allopurinol exposure was defined as no previous exposure in the baseline of 365 days

**Appendix 2.** Sensitivity analysis 3: Main models run using a different set of ICD-9 codes for Ventricular arrhythmias* based on Hennessey et al. [38]

|  | Multivariable-adjusted (Model 7) | | Multivariable-adjusted (Model 8) | |
| --- | --- | --- | --- | --- |
|  | HR (95% CI) | P-value | HR (95% CI) | P-value |
| Age (in years) |  |  |  |  |
| 65 - <75 | Ref |  | Ref |  |
| 75 - <85 | 1.04 (0.94, 1.15) | 0.46 | 1.04 (0.94, 1.15) | 0.45 |
| ≥85 | 1.08 (0.95, 1.23) | 0.25 | 1.08 (0.95, 1.24) | 0.24 |
| Gender |  |  |  |  |
| Male | Ref |  | Ref |  |
| Female | **0.74 (0.67, 0.81)** | **<0.0001** | **0.74 (0.67, 0.97)** | **<0.0001** |
| Race |  |  |  |  |
| White | Ref |  | Ref |  |
| Black | **1.27 (1.12, 1.43)** | **0.0002** | **1.26 (1.11, 1.43)** | **0.0003** |
| Other | **0.81 (0.68, 0.97)** | **0.023** | **0.78 (0.62, 0.98)** | **0.020** |
|  |  |  |  |  |
| Diuretics | 0.93 (0.75, 1.15) | 0.49 | 0.93 (0.75, 1.14) | 0.48 |
| Statins | **0.78 (0.62, 0.98)** | **0.034** | **0.78 (0.62, 0.98)** | **0.031** |
| ACE inhibitor | 1.00 (0.77, 1.28) | 0.97 | 1.00 (0.77, 1.28) | 0.97 |
| Beta blockers | 1.19 (0.97, 1.47) | 0.49 | 1.19 (0.97, 1.46) | 0.09 |
|  |  |  |  |  |
| Aspirin | 1.15 (0.29, 4.64) | 0.84 | 1.14 (0.28, 4.61) | 0.85 |
| Digoxin | **1.83 (1.33, 2.53)** | **0.0002** | **1.83 (1.33, 2.52)** | **0.0002** |
| Calcium channel blockers | 1.30 (0.76, 2.23) | 0.34 | 1.30 (0.76, 2.22) | 0.34 |
| Amiodarone | 0.83 (0.46, 1.49) | 0.52 | 0.83 (0.46, 1.49) | 0.52 |
| Flecainide | **0.00 (0.00, 0.00)** | **<0.0001** | **0.00 (0.00, 0.00)** | **<0.0001** |
| Ranolazine | 1.93 (0.72, 5.18) | 0.33 | 1.95 (0.73, 5.23) | 0.18 |
|  |  |  |  |  |
| Coronary artery disease | **2.19 (1.91, 2.51)** | **<0.0001** | **2.18 (1.90, 2.50)** | **<0.0001** |
| Cardiomyopathy- dilated or hypertrophic | **2.01 (1.80, 2.25)** | **<0.0001** | **2.09 (1.80, 2.24)** | **<0.0001** |
| Heart failure | **1.80 (1.62, 2.00)** | **<0.0001** | **1.80 (1.62, 2.00)** | **<0.0001** |
| Congenital heart disease | **1.48 (1.07, 2.05)** | **0.02** | **1.48 (1.07, 2.05)** | **0.020** |
| Valvular heart disease | **1.46 (1.30, 1.64)** | **<0.0001** | **1.45 (1.29, 1.64)** | **<0.0001** |
| Renal failure | **1.97 (1.75, 2.21)** | **<0.0001** | **1.97 (1.75, 2.21)** | **<0.0001** |
| Dialysis | 1.42 (0.99, 2.05) | 0.06 | 1.42 (0.98, 2.05) | 0.06 |
| Sarcoidosis | 1.03 (0.49, 2.18) | 0.94 | 1.03 (0.49, 2.18) | 0.94 |
| Hyperkalemia | **1.29 (1.15, 1.44)** | **<0.0001** | **1.29 (1.15, 1.40)** | **<0.0001** |
|  |  |  |  |  |
| Allopurinol use | **0.81 (0.74, 0.90)** | **<0.0001** | - | - |
| Allopurinol use duration^†^ |  |  |  |  |
| 0 days | - | - | Ref |  |
| 1-180 days | - | - | 0.93 (0.82, 1.06) | 0.28 |
| 181 days -2 years | - | - | **0.76 (0.67, 0.86)** | **<0.0001** |
| > 2 years | - | - | **0.72 (0.58, 0.90)** | **0.004** |

*Used a difference set of ICD-9 codes for this sensitivity analysis: 427.1, 427.2, 427.4, 427.41, 427.42, 427.5, 798, 798.1, and 798.2, based on the Hennessey paper [38], instead of 427.1, 427.2, 427.4x, 427.5, 427.60, or 427.69

HR, Hazard ratio; CI, confidence interval; Ref, referent category

^†^based on person day count

Model 7 = Model 5 re-run with this new set of ICD-9 codes (Allopurinol Use + age + race + gender + beta blockers + diuretics + ACE inhibitors + Statins + CAD + cardiomyopathy + heart failure + Congenital heart disease + Valvular heart disease + renal failure + Dialysis + sarcoidosis + hyperkalemia + aspirin + Digoxin + calcium channel blockers + Amiodarone + Flecainide + Ranolazine)

Model 8 = Model 6 re-run with this new set of ICD-9 codes (Allopurinol duration + age + race + gender + beta blockers + diuretics + ACE inhibitors + Statins + CAD + cardiomyopathy + heart failure + Congenital heart disease + Valvular heart disease + renal failure + Dialysis + sarcoidosis + hyperkalemia + aspirin + Digoxin + calcium channel blockers + Amiodarone + Flecainide + Ranolazine)

**Appendix 3.** Sensitivity analysis 4: Association of risk factors with hazard of ventricular arrhythmias in patients who received allopurinol including specific disease risk factors and three additional anti-arrhythmic medications* (mexilitine, propafenone and dofetilide)

|  | Univariate | | Multivariable-adjusted (Model 9) | | Multivariable-adjusted  (Model 10) | |
| --- | --- | --- | --- | --- | --- | --- |
|  | HR (95% CI) | P-value | HR (95% CI) | P-value | HR (95% CI) | P-value |
| Age (in years) |  |  |  |  |  |  |
| 65 - <75 | Ref |  | Ref |  | Ref |  |
| 75 - <85 | **1.25**  **(1.15, 1.36)** | **<0.0001** | 1.04  (0.94, 1.15) | 0.46 | 1.04  (0.94, 1.15) | 0.45 |
| ≥85 | **1.43**  **(1.28, 1.60)** | **<0.0001** | 1.08  (0.95, 1.23) | 0.25 | 1.08  (0.95, 1.23) | 0.24 |
| Gender |  |  |  |  |  |  |
| Male | Ref |  | Ref |  | Ref |  |
| Female | 0.81  (0.75, 0.87) | 0.50 | **0.74**  **(0.67, 0.81)** | **<0.0001** | **0.74**  **(0.67, 0.81)** | **<0.0001** |
| Race |  |  |  |  |  |  |
| White | Ref |  | Ref |  | Ref |  |
| Black | **1.32**  **(1.19, 1.48)** | **<0.0001** | **1.27**  **(1.12, 1.43)** | **0.0002** | **1.26**  **(1.11, 1.43)** | **0.0003** |
| Other | **0.80**  **(0.69, 0.94)** | **0.005** | **0.81**  **(0.68, 0.97)** | **0.023** | **0.81**  **(0.67, 0.97)** | **0.02** |
|  |  |  |  |  |  |  |
| Diuretics | 1.12  (0.94, 1.33) | 0.21 | 0.93  (0.75, 1.15) | 0.49 | 0.93  (0.75, 1.14) | 0.48 |
| Statins | 0.86  (0.70, 1.05) | 0.13 | **0.78**  **(0.62, 0.98)** | **0.034** | **0.78**  **(0.62, 0.98)** | **0.031** |
| ACE inhibitor | 1.09  (0.88, 1.34) | 0.43 | 0.93  (0.75, 1.15) | 0.49 | 0.99  (0.77, 1.28) | 0.97 |
| Beta blockers | **1.42**  **(1.20, 1.68)** | **<0.0001** | 1.19  (0.97, 1.47) | 0.09 | 1.19  (0.97, 1.46) | 0.09 |
|  |  |  |  |  |  |  |
| Aspirin | 1.04  (0.26, 4.18) | 0.95 | 1.15  (0.29, 4.63) | 0.85 | 1.14  (0.28, 4.61) | 0.85 |
| Digoxin | **2.93**  **(2.20, 3.91)** | **<0.0001** | **1.82**  **(1.32, 2.52)** | **0.0003** | **1.82**  **(1.32, 2.52)** | **0.0003** |
| Calcium channel blockers | 1.12  (0.90, 1.39) | 0.33 | 1.30  (0.76, 2.23) | 0.34 | 1.30  (0.76, 2.23) | 0.34 |
| Amiodarone | 1.12  (0.88, 1.43) | 0.36 | 0.83  (0.46, 1.49) | 0.53 | 0.83  (0.46,1.49) | 0.53 |
| Flecainide | 1.36  (0.19, 9.60) | 0.76 | **0.00**  **(0.00, 0.00)** | **<0.0001** | **0.00**  **(0.00, 0.00)** | **<0.0001** |
| Ranolazine | **2.97**  **(1.11, 7.94)** | **0.030** | 1.93  (0.72, 5.18) | 0.19 | 1.95  (0.73, 5.23) | 0.18 |
|  |  |  |  |  |  |  |
| Coronary artery disease | **3.34**  **(3.02, 3.71)** | **<0.0001** | **2.19**  **(1.91, 2.50)** | **<0.0001** | **2.18**  **(1.91, 2.50)** | **<0.0001** |
| Cardiomyopathy- dilated or hypertrophic | **3.84**  **(3.52, 4.19)** | **<0.0001** | **2.02**  **(1.81, 2.25)** | **<0.0001** | **2.01**  **(1.80, 2.24)** | **<0.0001** |
| Heart failure | **2.89**  **(2.67, 3.12)** | **<0.0001** | **1.80**  **(1.62, 2.00)** | **<0.0001** | **1.80**  **(1.62, 2.00)** | **<0.0001** |
| Congenital heart disease | **2.29**  **(1.69, 3.10)** | **<0.0001** | **1.48**  **(1.07, 2.05)** | **0.018** | **1.48**  **(1.07, 2.05)** | **0.019** |
| Valvular heart disease | **3.00**  **(2.71, 3.32)** | **<0.0001** | **1.46**  **(1.29, 1.65)** | **<0.0001** | **1.45**  **(1.29, 1.64)** | **<0.0001** |
| Renal failure | **2.87**  **(2.64, 3.12)** | **<0.0001** | **1.97**  **(1.75, 2.21)** | **<0.0001** | **1.97**  **(1.75, 2.21)** | **<0.0001** |
| Dialysis | **2.97**  **(2.14, 4.12)** | **<0.0001** | 1.42  (0.99, 2.05) | 0.06 | 1.42  (0.98, 2.04) | 0.06 |
| Sarcoidosis | 1.12  (0.54, 2.36) | 0.76 | 1.02  (0.48, 2.16) | 0.96 | 1.02  (0.48, 2.16) | 0.96 |
| Hyperkalemia | **2.31**  **(2.11, 2.54)** | **<0.0001** | **1.29 (1.15, 1.44)** | **<0.0001** | **1.28**  **(1.15, 1.43)** | **<0.0001** |
|  |  |  |  |  |  |  |
| Mexilitine | 17.88  (2.75, 116) | 0.003 | **0.00**  **(0.00, 0.00)** | **<0.0001** | **0.00**  **(0.00, 0.00)** | **<0.0001** |
| Propafenone | 0.00 (0.00,0.00) | <0.0001 | **14.08**  **(2.27, 87.1)** | **0.005** | **14.09**  **(2.33, 85.2)** | **0.004** |
| Dofetilide | 0.001 (0.00,0.00) | <0.0001 | **0.00**  **(0.00, 0.00)** | **<0.0001** | **0.00**  **(0.00, 0.00)** | **<0.0001** |
|  |  |  |  |  |  |  |
| Allopurinol use | **0.86**  **(0.79, 0.94)** | **0.001** | **0.81**  **(0.74, 0.90)** | **<0.0001** | - | - |
| Allopurinol use duration^†^ |  |  |  |  |  |  |
| 0 days | Ref |  | - | - | Ref |  |
| 1-180 days | 0.99  (0.88, 1.12) | 0.88 | - | - | 0.93  (0.81, 1.06) | 0.27 |
| 181 days -2 years | **0.81**  **(0.72, 0.90)** | **0.0002** | - | - | **0.76**  **(0.67, 0.86)** | **<0.0001** |
| > 2 years | **0.74**  **(0.62, 0.90)** | **0.002** | - | - | **0.72**  **(0.58, 0.90)** | **0.004** |

HR, Hazard ratio; CI, confidence interval; Ref, referent category

*Considered other anti- arrhythmics including quinidine, procainamide, but low frequency of use precluded their inclusion in the model.

^†^based on person day count

Model 9 = Model 7 with three additional anti-arrhythmic medications (Allopurinol Use + age + race + gender + beta blockers + diuretics + ACE inhibitors + Statins + CAD + cardiomyopathy + heart failure + Congenital heart disease + Valvular heart disease + renal failure + Dialysis + sarcoidosis + hyperkalemia + aspirin + Digoxin + calcium channel blockers + Amiodarone + Flecainide + Ranolazine + Mexilitine + Propafenone + Dofetilide

Model 10 = Model 8 with three additional anti-arrhythmic medications (Allopurinol duration + age + race + gender + beta blockers + diuretics + ACE inhibitors + Statins + CAD + cardiomyopathy + heart failure + Congenital heart disease + Valvular heart disease + renal failure + Dialysis + sarcoidosis + hyperkalemia + aspirin + Digoxin + calcium channel blockers + Amiodarone + Flecainide + Ranolazine + Mexilitine + Propafenone + Dofetilide)

**Appendix 4**. Subgroup Analysis by prior MI: Allopurinol use and duration of allopurinol use models by prior MI diagnosis

|  | Allopurinol use  HR (95% CI) [p-value] | | Allopurinol use duration  HR (95% CI) [p-value] | | | |
| --- | --- | --- | --- | --- | --- | --- |
|  | No | Yes | Non-use | 1-180 days | 181 days -2 years | >2 years |
| MI | Using model 1 | | Using model 2 | | | |
| No | Ref | **0.86 (0.78, 0.95) [p=0.003]** | Ref | 0.99 (0.86, 1.14) [p=0.85] | **0.82 (0.72, 0.93) [p=0.003]** | **0.70 (0.56, 0.88) [p=0.002]** |
| Yes | Ref | 0.78 (0.58, 1.05) [p=0.10] | Ref | 1.06 (0.71, 1.61) [p=0.77] | 0.68 (0.45, 1.02) [p=0.06] | **0.41 (0.18, 0.94) [p=0.03]** |
| MI | Using model 5 | | Using model 6 | | | |
| No | Ref | **0.82 (0.74, 0.91) [p=0.0002]** | Ref | 0.93 (0.80, 1.07) [p=0.29] | **0.77 (0.68, 0.88) [p<0.0001]** | **0.76 (0.60, 0.95) [p=0.02]** |
| Yes | Ref | 0.75 (0.56, 1.01) [p=0.06] | Ref | 0.97 (0.65, 1.46) [p=0.88] | 0.66 (0.44, 1.00) [p=0.05] | **0.43 (0.19, 0.96)** **[p=0.04]** |

Model 1 = Allopurinol Use+ age + race + gender + Charlson-Romano score + beta blockers + diuretics + ACE inhibitors + Statins

Model 5 = Allopurinol Use + age + race + gender + beta blockers + diuretics + ACE inhibitors + Statins + CAD + cardiomyopathy + heart failure + Congenital heart disease + Valvular heart disease + renal failure + Dialysis + sarcoidosis + hyperkalemia + aspirin + digoxin + calcium channel blockers + amiodarone + flecainide + ranolazine

**Appendix 5.** Hazard ratios of Ventricular arrhythmias by race and gender, adjusted for other factors

|  | Model 1 including allopurinol use | | Model 2 including allopurinol duration | |
| --- | --- | --- | --- | --- |
|  | HR (95% CI) | p-value | HR (95% CI) | p-value |
| **Male** | - |  |  |  |
| Black vs. Other | **1.48 (1.15, 1.91)** | **0.002** | **1.48 (1.15, 1.91)** | **0.002** |
| Black vs. White | **1.21 (1.02, 1.43)** | **0.03** | **1.20 (1.02, 1.42)** | **0.03** |
| White vs. Other | 0.81 (0.66, 1.00) | 0.05 | **0.81 (0.66, 0.996)** | **0.04** |
| **Female** |  |  |  |  |
| Black vs. Other | **1.34 (1.04, 1.73)** | **0.02** | **1.34 (1.04, 1.73)** | **0.02** |
| Black vs. White | **1.27 (1.09, 1.47)** | **0.002** | **1.27 (1.09, 1.47)** | **0.002** |
| White vs. Other | 0.95 (0.75, 1.19) | 0.64 | 0.94 (0.75, 1.18) | 0.61 |
|  |  |  |  |  |
| **Black** |  |  |  |  |
| Female vs. Male | **0.78 (0.63, 0.95)** | **0.02** | **0.78 (0.63, 0.95)** | **0.01** |
| **White** |  |  |  |  |
| Female vs. Male | **0.74 (0.67, 0.81)** | **<0.0001** | **0.73 (0.67, 0.81)** | **<0.0001** |
| **Other race** |  |  |  |  |
| Female vs. Male | 0.86 (0.64, 1.15) | 0.31 | 0.86 (0.64, 1.15) | 0.31 |

Model 1 = Allopurinol Use + age + race + gender + beta blockers + diuretics + ACE inhibitors + Statins + CAD + cardiomyopathy + heart failure + Congenital heart disease + Valvular heart disease + renal failure + Dialysis + sarcoidosis + hyperkalemia + aspirin + digoxin + calcium channel blockers + amiodarone + flecainide + ranolazine + race*gender

Model 2 = Allopurinol duration + age + race + gender + beta blockers + diuretics + ACE inhibitors + Statins + CAD + cardiomyopathy + heart failure + Congenital heart disease + Valvular heart disease + renal failure + Dialysis + sarcoidosis + hyperkalemia + aspirin + digoxin + calcium channel blockers + amiodarone + flecainide + ranolazine + race*gender

**Appendix 6.** Association of risk factors with hazard of **ventricular fibrillation** in patients who received allopurinol with no baseline ventricular fibrillation before the index date of allopurinol episode

|  | Univariate | | Multivariable-adjusted (model 1) | | Multivariable-adjusted  (Model 2) | |
| --- | --- | --- | --- | --- | --- | --- |
|  | HR (95% CI) | P-value | HR (95% CI) | P-value | HR (95% CI) | P-value |
| Age (in years) |  |  |  |  |  |  |
| 65 - <75 | Ref |  | Ref |  | Ref |  |
| 75 - <85 | 0.97  (0.74, 1.27) | 0.84 | 0.88  (0.67, 1.15) | 0.34 | 0.88  (0.67, 1.15) | 0.34 |
| ≥85 | 0.90  (0.60, 1.33) | 0.58 | 0.78  (0.53, 1.15) | 0.22 | 0.78  (0.53, 1.15) | 0.22 |
| Gender |  |  |  |  |  |  |
| Male | Ref |  | Ref |  | Ref |  |
| Female | 0.84  (0.66, 1.09) | 0.19 | 0.86  (0.67, 1.11) | 0.25 | 0.86  (0.67, 1.11) | 0.24 |
| Race |  |  |  |  |  |  |
| White | Ref |  | Ref |  | Ref |  |
| Black | 0.73  (0.47, 1.13) | 0.16 | 0.65  (0.42, 1.01) | 0.55 | 0.65  (0.42, 1.01) | 0.05 |
| Other | 0.74  (0.45, 1.21) | 0.23 | 0.73  (0.45, 1.20) | 0.22 | 0.73  (0.45, 1.20) | 0.22 |
| Charlson-Romano score, per unit change | **1.18**  **(1.14, 1.21)** | **<0.0001** | **1.18**  **(1.15, 1.21)** | **<0.0001** | **1.18**  **(1.15, 1.21)** | **<0.0001** |
| Diuretics | 1.04  (0.58, 1.88) | 0.89 | 0.96  (0.51, 1.83) | 0.91 | 0.96  (0.51, 1.83) | 0.91 |
| Statins | 0.83  (0.41, 1.68) | 0.60 | 0.75  (0.35, 1.60) | 0.46 | 0.75  (0.35, 1.60) | 0.46 |
| ACE inhibitor | 1.04  (0.52, 2.08) | 0.91 | 1.07  (0.53, 2.20) | 0.84 | 1.07  (0.53, 2.19) | 0.84 |
| Beta blockers | 1.41  (0.81, 2.46) | 0.22 | 1.42  (0.79, 2.54) | 0.24 | 1.42  (0.79, 2.54) | 0.24 |
| Allopurinol use | 0.93  (0.71, 1.22) | 0.59 | 0.89  (0.68, 1.16) | 0.39 | - | - |
| Allopurinol use duration* |  |  |  |  |  |  |
| 0 days | Ref |  | - | - | Ref |  |
| 1-180 days | 0.92  (0.61, 1.38) | 0.68 | - | - | 0.90  (0.60, 1.35) | 0.60 |
| 181 days -2 years | 1.00  (0.72, 1.40) | 0.99 | - | - | 0.94  (0.67, 1.32) | 0.74 |
| > 2 years | 0.76  (0.43, 1.33) | 0.33 | - | - | 0.73  (0.41, 1.28) | 0.27 |

HR, Hazard ratio; CI, confidence interval; Ref, referent category

*based on person day count

Model 1 = Allopurinol Use+ age + race + gender + Charlson-Romano score + beta blockers + diuretics + ACE inhibitors + Statins

Model 2 = Allopurinol duration + age + race + gender + Charlson-Romano score + beta blockers + diuretics + ACE inhibitors + Statins

Appendix 7. Sensitivity Analysis by underlying diagnosis of gout vs. non-gout: Association of allopurinol with hazard of ventricular arrhythmias adjusted for specific disease risk factors for ventricular arrhythmias

|  | Non-gout | | Gout | |
| --- | --- | --- | --- | --- |
|  | HR (95% CI) | P-value | HR (95% CI) | P-value |
|  | Multivariable-adjusted (Model 3) | | | |
| Allopurinol use | 0.89 (0.71, 1.10) | 0.28 | **0.81 (0.73, 0.90)** | **0.0001** |
|  | Multivariable-adjusted (Model 4) | | | |
| Allopurinol use duration* |  |  |  |  |
| 0 days (non-use) | - | - | Ref |  |
| 1-180 days | 0.95 (0.68, 1.32) | 0.75 | 0.94 (0.81, 1.09) | 0.41 |
| 181 days -2 years | 0.82 (0.61, 1.10) | 0.19 | **0.76 (0.66, 0.87)** | **<0.0001** |
| > 2 years | 0.95 (0.52, 1.71) | 0.85 | **0.68 (0.54, 0.87)** | **0.002** |
| HR, Hazard ratio; CI, confidence interval; Ref, referent category  *based on person day count  Model 3 = **Allopurinol Use** + age + race + gender + beta blockers + diuretics + ACE inhibitors + Statins + CAD + cardiomyopathy + heart failure + congenital heart disease + valvular heart disease + renal failure + Dialysis + sarcoidosis + hyperkalemia  Model 4 = **Allopurinol duration** + age + race + gender + beta blockers + diuretics + ACE inhibitors + Statins + CAD + cardiomyopathy + heart failure + congenital heart disease + valvular heart disease + renal failure + dialysis + sarcoidosis + hyperkalemia  Significant hazards ratios and p-values are **in bold** | | | | |

Appendix 8. Sensitivity Analysis limiting the cohort to patients not receiving any **anti-arrythmic or cardio-protective drugs**: Association of allopurinol with hazard of ventricular arrhythmias adjusted for specific disease risk factors for ventricular arrhythmias

|  | Multivariable-adjusted (model 1) | | Multivariable-adjusted (Model 2) | |
| --- | --- | --- | --- | --- |
|  | HR (95% CI) | P-value | HR (95% CI) | P-value |
| Allopurinol use | **0.82 (0.75, 0.91)** | **0.0002** | - | - |
| Allopurinol use duration* |  |  |  |  |
| 0 days (non-use) | - | - | Ref |  |
| 1-180 days |  |  | 0.96 (0.83, 1.11) | 0.58 |
| 181 days -2 years |  |  | **0.76 (0.67, 0.87)** | **<0.0001** |
| > 2 years |  |  | **0.74 (0.60, 0.93)** | **0.008** |
